# Epidemiology of Travel-associated Pandemic (H1N1) 2009 Infection in 116 Patients, Singapore

**DOI:** 10.3201/eid1601.091376

**Published:** 2010-01

**Authors:** Pratik Mukherjee, Poh Lian Lim, Angela Chow, Timothy Barkham, Eillyne Seow, Mar Kyaw Win, Arlene Chua, Yee Sin Leo, Mark I-Cheng Chen

**Affiliations:** Tan Tock Seng Hospital, Singapore (P. Mukherjee, P.L. Lim, A. Chow, T. Barkham, E. Seow, M.K. Win, A. Chua, Y.S. Leo, M.I-C. Chen); National University of Singapore, Singapore (A. Chow, M.I-C. Chen); Duke–National University of Singapore Graduate Medical School, Singapore (M.I-C. Chen)

**Keywords:** Influenza A virus, pandemic (H1N1) 2009, travel, influenza, pandemic, disease transmission, viruses, Singapore, expedited, research

## Abstract

In June 2009, during Singapore’s pandemic influenza plan containment phase, pandemic (H1N1) 2009 was introduced into the country through imported cases. To understand how travel patterns affected the initial outbreak, we examined epidemiologic and travel data for the first 116 case-patients admitted to Tan Tock Seng Hospital, Singapore, with travel-associated infection. Sixty-one percent and 54% of patients, respectively, met US Centers for Disease Control and Prevention and World Health Organization temperature criteria for influenza-like illness. One fourth of the case-patients traveled after illness onset, and 15% became ill while traveling. Regions of exposure for imported infections changed rapidly; case-patients initially arrived from North America, followed by Australasia and Southeast Asia. Case-patients on longer flights were more likely to become ill before arrival; those with shorter flights tended to become ill after arrival. Thermal scanners detected fevers in 12% of the arriving case-patients, resulting in a shorter time to isolation.

## Abstract 2

During the containment phase, regions of exposure for imported infections changed rapidly.

On April 24, 2009, international authorities reported cases of infection with a novel influenza A virus (H1N1) strain of swine origin, now known as pandemic (H1N1) 2009 virus; 7 cases in the United States and 3 clusters in Mexico were confirmed, and surveillance indicated influenza-like-illness (ILI) had been increasing in Mexico since March 18, 2009 (*1*). During the next 3 months, this virus spread rapidly across the globe, resulting in >254,206 cases and at least 2,837 deaths on 6 continents as of August 30, 2009 (*2*). For most countries, the initial introduction of this virus occurred through international travel and human-to-human transmission.

The role of air travel in the transmission and dissemination of respiratory infections has been examined for severe acute respiratory syndrome (SARS), pneumonic plague, and extensively drug-resistant tuberculosis (*3*–*6*). Grais et al. (*7*) explored the possible effect of airline travel on geographic spread of pandemic influenza in 2000 through simulation models of the pandemic influenza virus (H3N2) of 1968–69, using air travel data for 53 global cities. The effect of air travel on facilitating the transmission and dissemination of influenza is borne out by other recent studies suggesting that decreased volumes of air travel in the 2–3 months after the terrorist attacks in the United States on September 11, 2001, delayed that winter’s seasonal influenza peak and decreased transmission (*8*).

Human-to-human transmission of influenza during air travel has been reported to occur on flights of at least 8 hours and to affect passengers seated within 2 rows of the index case-patient (*6*). However, other reports show that respiratory infections can spread during shorter flights and over considerable distances within the cabin (*3*,*9*). For the purposes of influenza contact tracing, the World Health Organization (WHO) has defined considerable exposure on aircraft as being restricted to passengers sitting in the same row or within 2 rows of an infectious person for a flight time of >8 hours (*10*).

Singapore implemented the containment phase of its pandemic influenza plan on April 27, 2009, before pandemic (H1N1) 2009 was introduced into the country. Public health policy during this phase was to isolate infected case-patients and quarantine exposed persons to prevent local transmission for as long as possible. During this initial period, travel from a pandemic (H1N1) 2009–affected area was the major risk factor for infection; air travel was the main route of introduction. The containment phase was in effect for >2 months until epidemiologic surveillance indicated sustained community spread had begun during epidemiologic week 25 (week ending June 27, 2009). At that time, a gradual transition to mitigation measures was implemented.

During the containment phase, airport thermal scanners were used to detect fevers in arriving passengers at Singapore’s Changi International Airport, and health advisories were used to encourage travelers in whom influenza-like symptoms developed after disembarkation to seek medical care. Ambulances, dedicated for this purpose only, were used to transport suspected case-patients from the airport to hospitals for screening. Adults with appropriate travel histories and ILI were referred to the designated screening center at Tan Tock Seng Hospital (TTSH) for treatment and isolation at the Communicable Disease Centre. During the mitigation phase, the focus of clinical and public health efforts shifted to caring for patients with severe illness and conditions that put them at risk for complications and to reducing transmission in the community through health education and voluntary self-isolation of persons with ILI.

We analyzed epidemiologic and travel data as well as data regarding source of referral for case-patients in relation to time of illness onset, time of arrival in Singapore, and time to isolation. Understanding how travel patterns affected propagation of the first pandemic wave of pandemic (H1N1) 2009 virus could yield insights into how the anticipated next wave might be disseminated and provide data on the effectiveness and limitations of different interventions used to slow dissemination.

## Methods

We obtained demographic data and travel-related information (last port of embarkation and flight times) by direct interview and retrospective review of clinical notes. Duration of travel, which was calculated from flight times and transit time within airports, was categorized into 4 groups: short haul (<3 hours), medium haul (3–6 hours), long haul (6–15 hours), and extra-long haul (>15 hours). Clinical data were collected for symptoms and date and time of symptom onset (to the nearest hour in most instances and to the nearest 8-hour block [midnight to 7:59 am, 8:00 am to 3.59 pm, or 4:00 pm to 11:59 pm] if the patient was unsure). Symptom onset time was further categorized as before embarkation, during travel, or after disembarkation in Singapore. We also categorized hospitalized case-patients on the basis of whether their symptoms met US Centers for Disease Control and Prevention (CDC) ILI criteria (body temperature >37.8°C with either cough or sore throat in the absence of an alternative diagnosis) and WHO ILI criteria (body temperature >38°C with either cough or sore throat in the absence of an alternative diagnosis) (*11*,*12*).

A confirmed case of pandemic (H1N1) 2009 was defined as an ILI in a patient with a temperature >37.5°C, cough, rhinorrhea, sore throat, or myalgia and with laboratory confirmation of infection by real-time reverse transcription–PCR performed on respiratory samples (sputum or combined nasal and throat swab specimens). An imported, travel-associated case was defined as above but occurred in a person with recent travel outside Singapore who had arrived in Singapore during the containment period and had illness onset within 10 days of arrival.

We compared proportions by using the χ^2^ test. Means were compared by use of *t* tests for dichotomous variables and 1-way analysis of variance for multichotomous variables. Stata 10.0 for Windows (StataCorp LP, College Station, TX, USA) was used for all statistical analyses.

## Results

From April 27 through June 27, 2009 (epidemiologic week 25), 152 persons with confirmed pandemic (H1N1) 2009 were admitted to TTSH. Of the 152 patients, 116 met the definitions for having an imported, travel-associated case; the rest either did not have a history of travel or had onset of symptoms >10 days after their travel ended. These 116 cases span 5 weeks (epidemiologic weeks 21–25) from when the first confirmed case-patient arrived in Singapore on May 26, 2009.

Demographic and travel data for patients with cases of imported pandemic (H1N1) 2009 are shown in Table 1. Infections involved equal numbers of male and female travelers and occurred predominantly in young travelers (mean age 28.5 years; 70% were <30 and 7% were >50 years of age). Half of the travelers were Singaporeans returning from abroad, and the other half were travelers from other countries.

**Table 1:** Demographic and travel-related data for 116 persons with travel-associated pandemic (H1N1) 2009, Singapore, April 26–June 27, 2009*

Data	Case-patients, no. (%)
Sex	
M	59 (50.9)
F	57 (49.1)
Age group, y	
<19	18 (15.5)
20–29	63 (54.3)
30–39	13 (11.2)
40–49	14 (12.1)
>50	8 (6.9)
Nationality	
Singaporean	58 (50.0)
Others	58 (50.0)
Port of embarkation, by region	
Americas	22 (19.0)
Asia	56 (48.3)
Australasia	31 (26.7)
Europe	7 (6.1)
Duration of travel, h	
<3	22 (19.0)
3–5.9	34 (29.3)
6–14.9	36 (31.0)
>15	24 (20.7)
Onset of symptoms	
Before embarkation	29 (25.0)
While travelling	17 (14.7)
After disembarkation	70 (60.3)
Source of referral to TTSH screening center	
Airport doctor	15 (12.9)
Community doctor	50 (43.1)
Self-referred	51 (44.0)
Screening criteria documented during clinical examination	
Temperature >37.5°C	84 (72.4)
Temperature >37.8°C	71 (61.2)
Temperature >38.0°C	61 (52.6)
US CDC ILI criteria†	60 (51.7)
WHO ILI criteria‡	51 (44.0)
Arrival at TTSH, by epidemiologic week§	
21	5 (4.3)
22	8 (6.9)
23	13 (11.2)
24	41 (35.3)
25	49 (42.2)

*TTSH, Tan Tock Seng Hospital, Singapore; US CDC, US Centers for Disease Control and Prevention; ILI, influenza-like illness; WHO, World Health Organization. †Temperature >37.8°C plus cough or sore throat. ‡Temperature >38.0°C plus cough or sore throat. §Week 21, May 24–30; week 22, May 30–June 6; week 23, June 7-13; week 24, June 14–20; week 25, June 21–27.

**Figure 1:**
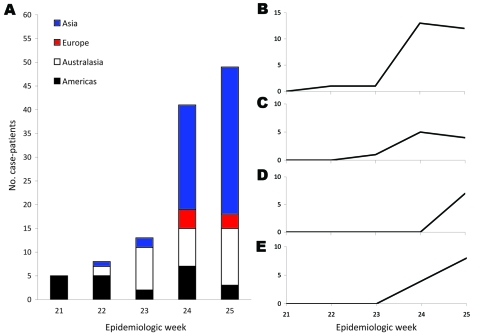
Sources of exposure, by region and country, among 116 patients in Singapore infected with pandemic (H1N1) 2009 virus identified during epidemiologic weeks 21–25, 2009. A) Asia compared with other regions; B) the Philippines; C) Thailand; D) Indonesia; E) other Asian countries. Week 21, May 24–30; week 22, May 30–June 6; week 23, June 7–13; week 24, June 14–20; week 25, June 21–27.

As shown in Figure 1, the regions where travelers were exposed to pandemic (H1N1) 2009 virus changed rapidly during the 5-week period of study. Most of the cases in epidemiologic weeks 21 and 22 (May 24 through June 6) were in patients who acquired their infection in the United States; however, within 2 weeks, this had changed dramatically, with a large proportion of cases coming from Australasia in epidemiologic week 23 (June 7–13). Within a week, this situation changed yet again; most infections originated from Southeast Asia by epidemiologic weeks 24–25 (June 14–27).

The first cases of pandemic (H1N1) 2009 detected among travelers arriving from the Philippines (week 22), Thailand (week 23), and Indonesia (week 25) indicated potential community transmission in those countries earlier than official announcements. Infections from Southeast Asian countries accounted for 29% of imported cases during the containment phase; the Philippines were linked to 23% cases, mostly in weeks 24–25.

Travel duration reflects the distance between the regions of exposure and Singapore (Table 1). Of the 116 case-patients, 19% had a short travel duration (<3 hours), and 20% had a long travel duration (>15 hours). Although most case-patients became ill after arrival at their destination, 25% were ill before travel and boarded their flight despite symptomatic illness, and 15% became ill while traveling.

Doctors based at the airport referred 15 (12.9%) of the 116 patients to TTSH; thermal scanners used to screen arriving passengers had detected fever in 14 of these 15 patients. Of the remaining 101 patients, 51 (44%) self-reported to the screening center at TTSH and 50 (43%) were referred by doctors in the community.

At the time of examination, 72% of case-patients had temperatures >37.5°C. However, only 61.2% had temperatures >37.8°C and 54% had temperatures >38°C, the temperature criteria used in the US CDC and WHO ILI case definitions, respectively. Considering the entire symptom complex, 51% of patients would have fulfilled ILI criteria as defined by the US CDC, and 44% would have fulfilled the WHO criteria (*11*,*12*).

**Figure 2:**
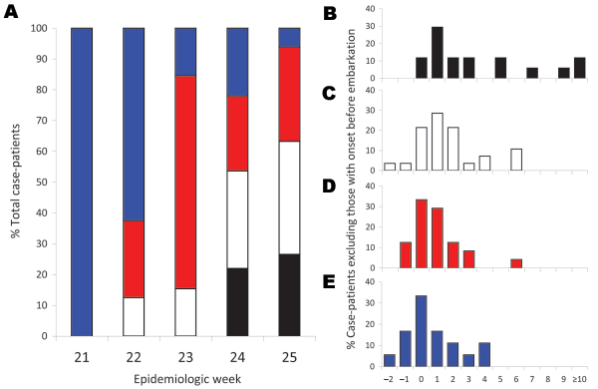
Travel duration and illness onset relative to arrival in Singapore for 116 patients infected with pandemic (H1N1) 2009 virus identified during epidemiologic weeks 21–25, 2009. A) Distribution of travel duration by epidemiologic week; B–E) timing of illness onset by travel duration in case-patients who did not have symptoms before embarkation (n = 87). Black, travel duration <3 h; white, 3–5.9 h; red, 6–14.9 h; blue, >15 h. Mean time from arrival to illness onset was 3.5 days (95% confidence interval [CI] 1.9–5.2), 1.7 days (95% CI 0.9–2.4), 1.0 days (95% CI 0.4–1.6), and 0.8 days (95% CI 0.0–1.5), respectively. The percentage of patients with symptom onset before arrival was 0%, 14%, 29%, and 33%, respectively. Week 21, May 24–30; week 22, May 30–June 6; week 23, June 7–13; week 24, June 14–20; week 25, June 21–27.

As the pandemic shifted toward Asian ports of embarkation, the number of case-patients with travel durations of <8 hours increased (Figure 2, panel A). The time of symptom onset relative to arrival in Singapore is shown in Figure 2, panel B; the figure does not include information for patients who were symptomatic before embarkation. Time of symptom onset was progressively closer to the time of arrival in Singapore for those arriving from longer distances (p = 0.001). Patients with longer travel durations were also more likely to have onset of symptoms before arrival (p = 0.04).

**Figure 3:**
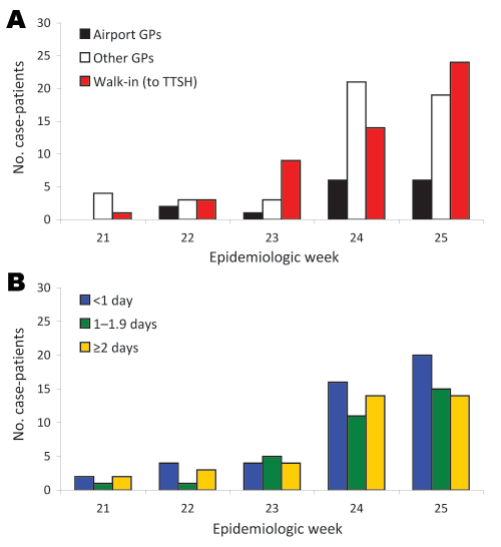
A) Source of referral to Tan Tock Seng Hospital (TTSH) and B) time to isolation for 116 patients infected with pandemic (H1N1) 2009 virus, Singapore. Week 21, May 24–30; week 22, May 30–June 6; week 23, June 7–13; week 24, June 14–20; week 25, June 21–27. GP, general practitioner.

Port of embarkation, clinical symptoms, and duration of travel did not predict delay to isolation (Table 2). However, case-patients referred to TTSS by airport doctors had a shorter time to isolation (0.76 days) than self-referred patients or those referred by other sources (1.6–1.9 days). The number of case-patients referred by airport doctors increased over the 5-week period, but they represent only 12% of all travel-associated cases (Figure 3). Although the mean duration to isolation did not increase significantly over the study period, total numbers of case-patients with delays to isolation increased as the volume of travel-associated cases rose.

**Table 2:** Predictors of time to isolation for 116 patients with travel-associated pandemic (H1N1) 2009, Singapore, April 26–June 27, 2009*

Predictive factor	No. patients	Delay to isolation, mean no. days (95% CI)†	p value‡
Port of embarkation			0.718
Asian countries	56	1.64 (1.32–1.96)	
All other countries	60	1.55 (1.20–1.91)	
Source of referral to TTSH			0.015
Airport doctors	15	0.76 (0.33–1.19)	
Community doctors	50	1.58 (1.28–1.88)	
Self-referral	51	1.85 (1.44–2.27)	
Met US-CDC ILI criteria§			0.426
No	56	1.69 (1.31–2.08)	
Yes	60	1.50 (1.21–1.79)	
Met WHO ILI criteria¶			0.650
No	65	1.64 (1.30–1.99)	
Yes	51	1.53 (1.21–1.86)	
Time of onset of symptoms			0.664
Before embarkation	29	1.78 (1.14–2.42)	
While traveling	17	1.49 (0.87–2.12)	
After disembarkation	70	1.54 (1.29–1.80)	
Duration of travel, h			0.350
<3	22	1.96 (1.37–2.55)	
3–5.9	34	1.39 (1.03–1.74)	
6–14.9	36	1.69 (1.31–2.08)	
>15	24	1.41 (0.74–2.08)	
Arrival at TTSH, by epidemiologic week#			0.868
21	5	1.83 (0.50–3.15)	
22	8	1.30 (0.44–2.16)	
23	13	1.87(1.13–2.61)	
24	41	1.61 (1.17–2.05)	
25	49	1.53 (1.19–1.87)	

*CI, confidence interval; TTSH, Tan Tock Seng Hospital, Singapore; US CDC, US Centers for Disease Control and Prevention; ILI, influenza-like illness; WHO, World Health Organization. †No. days from arrival to hospital examination in patients with onset before arrival and no. days from onset to hospital examination in patients with onset after arrival. ‡p value by *t* test for dichotomous variables and 1-way analysis of variance for multichotomous variables. §Temperature >37.8°C plus cough or sore throat. ¶Temperature >38.0°C plus cough or sore throat. #Week 21, May 24–30; week 22, May 30–June 6; week 23, June 7-13; week 24, June 14–20; week 25, June 21–27.

## Discussion

Given Singapore’s position as a major travel hub, with passenger traffic at Changi International Airport exceeding 37 million in 2008 (*13*), there is an ever-present risk of importation and subsequent community transmission of emerging respiratory infections. Such importation and transmission occurred during the SARS epidemic of 2003 (*14*) and remains a concern during the current pandemic of pandemic (H1N1) 2009 virus. In any epidemic, every imported case may start a cluster of locally transmitted cases, but the number of imported cases and delays to isolation are particularly important at the start of the epidemic in determining speed of spread within the community. Improving detection and shortening time to isolation of infectious persons could modify the outbreak curve and allow more time for improving community preparedness.

Improving detection and shortening the time to isolation of sick persons is the rationale for using airport thermal scanners. Our data show that for the minority of cases detected by airport thermal scanners, detection does result in a hospital referral by an airport doctor and shorter time to isolation. However, intrinsic limitations of airport thermal scanners are that passengers have to become symptomatic before disembarking from a flight and have a fever high enough to be detected. Our data show that >30% of case-patients from all flights >3 hours had symptom onset before arrival, but overall, only 12% of all case-patients were detected by thermal scanners, suggesting that thermal scanners detected 40% of those symptomatic patients. This early detection and isolation may still have a valuable adjunctive role, especially in the initial phase of outbreaks. Situations favoring the use of airport thermal scanners include short-incubation diseases and geographically distant outbreak epicenters, such that arriving passengers have been on a long-haul flight. However, if the converse were true, with transmission occurring in nearby countries and passengers arriving from short-haul flights, symptoms would develop in most passengers who become ill after entry and, thus, would be missed by airport thermal scanners.

The fact that one fourth of the case-patients in our study boarded a plane after becoming ill and traveled despite having symptoms illustrates the role of travelers in disseminating infection in a highly interconnected world. It raises the question of whether exit screening should be considered. However, the effectiveness of exit screening will depend on the role of asymptomatic persons in transmission, and such screening will still miss persons who are incubating the infection. Exit screening would severely hinder international travel, and because of its questionable efficacy, it may not be justified and may be contrary to the intent of the International Health Regulations 2005 (*15*)

Because 44% of the case-patients in this study were self-referred and 43% were referred by community physicians, prevention efforts should focus on other strategies. For example, health advisories should be distributed to arriving passengers, encouraging them to seek medical care if ill, and steps should be taken to improve detection by physicians who see patients in their clinics. The importance of clinical judgment, epidemiologic history, and access to diagnostic testing is emphasized by the substantial minority of case-patients who would not have met the WHO or US CDC ILI criteria but who did have laboratory-confirmed infection. However, our study did have limitations: clinical features were assessed at a single time point (on arrival at the hospital screening center) and there was potential recall bias.

Our data demonstrate how swiftly situations can change in a fast-moving pandemic; affected areas shifted from the Americas to Australasia to Asia within a matter of days. These rapid shifts pose a tremendous challenge to health authorities responsible for outbreak management, and they emphasize the narrow window of opportunity during which interventions can slow an epidemic. The monitoring of travelers fulfills a vital sentinel surveillance function, providing an early indicator of community transmission in countries even before transmission has been officially confirmed. Understanding how travel-associated infections propagated the first wave of this pandemic yields rich insights into how health authorities might respond to future outbreaks of emerging respiratory infections.
